# Syllabus of the Obstetrical Lectures in the Medical Department of the University of Pennsylvania

**Published:** 1894-06

**Authors:** 


### Book Reviews.

Syllabus of the Obstetrical Lectures in the Medical Department of the University of Pennsylvania. By Richard C. Norris, A.M., M.D., Demonstrator of Obstetrics, University of Pennsylvania; Assistant Obstetrician, University Maternity; Physician to the Methodist Episcopal Hospital; Obstetrical Registrar, Philadelphia Hospital; Consulting Obstetrician and Attending Gynecologist, Southeastern Dispensary and Hospital for Women and Children. Third Edition. Philadelphia: W. B. Saunders, 1894. Price $2.00.

In this, the third edition, the author has simply added some few points which were necessary to bring it up with recent investigations. This little book is written for the student and has been "prepared to meet the difficulty of accurate note-taking, which most medical students encounter." Except for the students of Dr. Norris the book cannot take the place of note-taking—but it is a valuable aid to any student for both the lecture room and the preparations for the green room. One side of each leaf is left blank for notes to be made by the student. V. H. T.

